# Generative Artificial Intelligence Use in Healthcare: Opportunities for Clinical Excellence and Administrative Efficiency

**DOI:** 10.1007/s10916-024-02136-1

**Published:** 2025-01-16

**Authors:** Soumitra S. Bhuyan, Vidyoth Sateesh, Naya Mukul, Alay Galvankar, Asos Mahmood, Muhammad Nauman, Akash Rai, Kahuwa Bordoloi, Urmi Basu, Jim Samuel

**Affiliations:** 1 Edward J. Bloustein School of Planning and Public Policy, Rutgers, The State University of New Jersey, 255, Civic Square Building 33 Livingston Ave #400, New Brunswick, NJ 08901 USA; 2 School of Social Policy, Rice University, Houston, TX USA; 3 Biotechnology High School, Freehold, NJ USA; 4 Center for Health System Improvement, College of Medicine, The University of Tennessee Health Science Center, Memphis, TN USA; 5 University of Engineering & Technology, Peshawar, Pakistan; 6 Department of Psychology and Counselling, St. Joseph’s University, Bangalore, India; 7 Insight Biopharma, Princeton, NJ USA

**Keywords:** Generative AI, Artificial intelligence, Healthcare, Large language models, Clinical excellence, Ethics, Health information technology, AI applications, ChatGPT, Medicine

## Abstract

Generative Artificial Intelligence (Gen AI) has transformative potential in healthcare to enhance patient care, personalize treatment options, train healthcare professionals, and advance medical research. This paper examines various clinical and non-clinical applications of Gen AI. In clinical settings, Gen AI supports the creation of customized treatment plans, generation of synthetic data, analysis of medical images, nursing workflow management, risk prediction, pandemic preparedness, and population health management. By automating administrative tasks such as medical documentations, Gen AI has the potential to reduce clinician burnout, freeing more time for direct patient care. Furthermore, application of Gen AI may enhance surgical outcomes by providing real-time feedback and automation of certain tasks in operating rooms. The generation of synthetic data opens new avenues for model training for diseases and simulation, enhancing research capabilities and improving predictive accuracy. In non-clinical contexts, Gen AI improves medical education, public relations, revenue cycle management, healthcare marketing etc. Its capacity for continuous learning and adaptation enables it to drive ongoing improvements in clinical and operational efficiencies, making healthcare delivery more proactive, predictive, and precise.

## Introduction

Artificial Intelligence (AI) is increasingly utilized across various fields, including healthcare, where it has the potential to revolutionize clinical decision-making and enhance operational efficiencies. A significant branch within AI, Generative AI (Gen AI), has recently attracted attention for its ability to create precise data through machine learning algorithms, such as text, images, and music [1, 2, 5]. AI has been defined as “a set of technologies that mimic the functions and expressions of human intelligence, specifically cognition, logic, learning, adaptivity, and creativity” [3]. Gen AI is characterized by its specialized capabilities to produce new content, data, or solutions based on patterns and information learned from existing datasets [4]. This capability arises from powerful foundation models that utilize advanced machine learning and deep learning techniques [6]. Foundation models are “large, complex models trained on vast quantities of digital general information that can later be adapted for more specific uses” [7]. These models feature two important characteristics: “emergence,” the propensity to develop new and often unexpected capabilities as they scale, and “homogenization,” the tendency to serve as a common intelligence base for varied specialized functions and AI applications [7]. The generative pre-trained transformer (GPT) model, particularly GPT-3 as a large language model (LLM) and GPT-4 as a large multi-modal model (LMM), exemplifies a foundation model [11, 12]. Other essential AI methods, such as generative adversarial networks (GANs) and variational autoencoders (VAEs), also play a critical role.

GANs involve a pair of neural networks—the generator and the discriminator—that work against each other. The generator strives to create data as realistically as possible. At the same time, the discriminator evaluates this data against real-world examples, iterating until the generated data is indistinguishable from actual data, at least to the discriminator [9, 10]. According to the market research firm Market.us, the global net value of Gen AI in healthcare was approximately $800 million in 2022, with projections to grow to $17.2 billion by 2032 [8]. The healthcare industry has embraced AI-powered solutions, allocating substantial funding to explore their practical benefits. A recent survey from the Deloitte Center for Health Solutions reveals that approximately 75% of large healthcare organizations are currently using or planning to scale up generative AI in their operations [13]. In 2023, Gen AI technologies made substantial advancements, as evidenced by a recent study in which ChatGPT surpassed physicians in both quality and empathy metrics [14], and Google’s MedPaLM-2 LLM achieved expert-level scores on the United States Medical Licensing Examination [15]. Furthermore, the first drug entirely designed with Gen AI entered clinical trials [15], while new GPUs are expected to reduce LLM costs in 2024 [16].

The healthcare industry is grappling with numerous systemic challenges, many of which have been intensified by the COVID-19 pandemic, affecting both workforce stability and patient care delivery. The pandemic has exacerbated labor shortages in healthcare, making workforce challenges a top concern for hospital CEOs [17]. The Association of American Medical Colleges predicts a shortfall of 124,000 physicians by 2034 in the US [18]. The industry also faces a shortage of 1.1 million nurses [19], resulting in longer waiting times to see a clinician. In 2019, the average wait time to see a physician was 26 days, with patients often making numerous phone calls to schedule visits [13]. Provider burnout is another significant concern, with 81% of the clinicians reporting high-stress levels primarily due to increased workloads and administrative burdens [20]. Clinicians have low trust in leadership, with fewer than 45% trusting their organization’s leadership to prioritize patient care [20]. Financially, the healthcare sector struggles with rising operational costs, shrinking reimbursement rates, and low profitability margins [21]. Despite increasing national healthcare expenditures, US life expectancy has declined recently, and healthcare accessibility remains a significant issue, especially in underserved communities [13]. Gen AI has the potential to address some of these challenges by improving healthcare efficiency, reducing administrative burdens, and enhancing patient care [22]. Given that Gen AI is a relatively new technology, literature is scarce on its clinical and non-clinical uses. This review article aims to explore the potential applications of Gen AI for clinical and non-clinical uses.

## Clinical Applications

### Tailored Treatment Plans

Generative AI has the potential to improve personalized medicine by analyzing extensive health datasets and identifying patterns for tailored treatment plans, thereby significantly enhancing patient outcomes. For instance, a research study demonstrated that deep learning models can predict cardiovascular risk factors from retinal images and pinpoint biomarkers for complex diseases, thus formulating precise, personalized treatment plans [25]. Using GANs to simulate virtual patient populations also helps predict treatment outcomes across diverse demographic and genetic backgrounds. Scientists have successfully utilized GANs to generate synthetic patient data, facilitating the creation of personalized treatment plans, especially for rare diseases where data is scarce or non-existent [26].

The Generative Tensorial Reinforcement Learning (GENTRL) model is particularly adept at designing drugs tailored to specific biological mechanisms, ensuring that drugs interact optimally with biological targets relevant to an individual patient’s condition [27]. This level of specificity significantly supports the development of personalized treatment plans. It also optimizes resources in genetic testing by streamlining workflows and automating tasks, making personalized medicine more accessible. Additionally, Gen AI enhances pharmacogenomic optimization by analyzing data to predict individual medication responses, leading to customized prescriptions and improved treatment outcomes [28]. Gen AI also processes wearable device data in real-time to monitor vital health indicators, facilitating early intervention and personalized treatment strategies [23].

In mental health, Gen AI creates interactive tools for cognitive behavioral therapy (CBT) [24]. The TP-GAN (treatment planning with GAN) framework, a cGANs-based model, represents a significant advancement in the automation of prostate brachytherapy planning. By reducing the planning time and potentially the variability associated with manual planning, this approach can lead to more standardized treatment plans and improve overall treatment efficacy [29]. AI algorithms also assist in diagnosing dental health conditions such as caries and periodontal diseases through image analysis and data interpretation, leading to timely interventions, personalized treatment plans, and enhanced treatment outcomes [30]. The implications of Gen AI for personalized medicine are vast, as evidenced by recent studies exploring its role in improving the diagnosis and management of rare genetic disorders. These technologies analyze large datasets for accurate classification and employ facial recognition to aid in diagnosing genetic conditions [31].

### Surgical Care

Surgical teams are increasingly leveraging AI to enhance surgical procedures. Before surgery, surgeons must synthesize medical literature with comprehensive patient data, including imaging, medical history, and relevant laboratory results, while adhering to the most recent treatment guidelines and protocols [32]. Gen AI models can integrate, analyze, and interpret these complex datasets, expediting surgical decision-making and improving the timeliness of treatment [33]. Since patients’ understanding of surgical procedures is crucial, Gen AI can play a vital role in educating patients about surgical procedures and associated risks, both pre- and post-surgery [34]. Additionally, Gen AI can be used for quality assurance, to detect errors and suggest new policies to improve patient care and ultimately improve health outcomes [35].

A notable collaboration between Johnson & Johnson’s MedTech unit and Nvidia has been established to enhance surgical processes by integrating AI from pre- to post-operative stages. This initiative uses AI to analyze surgical videos and automate the extensive documentation required after surgery [36]. Other surgical applications include the introduction of real-time AI annotation in urologic robotic surgeries to enhance surgical training and quality metrics. This technology aims to automatically annotate surgical videos, providing real-time feedback and support to surgeons and trainees, thereby increasing the safety, efficiency, and educational value of surgical procedures [37]. AI also has the potential to augment surgical decision-making through personalized risk assessments [38]. In recent years, minimally invasive surgical techniques such as laparoscopic surgery (LS) and robotic surgery (RS) have become increasingly prevalent. The ultimate goal of RS development is the creation of fully autonomous AI-powered surgical instruments. Although current systems require human supervision, their success in procedures like prostatectomies (Da Vinci system) and bowel suturing (STAR robot) showcases the potential of AI in enhancing surgical outcomes [39].

### Provider Burnout

Provider burnout is one of the major challenges for healthcare systems worldwide. The Association of American Medical Colleges (AAMC) declared physician burnout a public health crisis [40]. According to a 2019 study, more than half of US physicians have experienced at least one common burnout symptom, such as a reduced sense of personal accomplishment, emotional exhaustion, depersonalization, and the inability to connect with family members. As the same study reports, burnouts substantially negatively impact the US economy, costing approximately $4.6 billion annually [41]. Gen AI tools have shown promise in reducing physician burnout and enhancing efficiency. Large language models, such as generative pre-trained transformer 4 (GPT-4; OpenAI), have shown the ability to draft empathetic responses to online patient questions [42].

A recent study found that using GPT-4 to generate draft replies to patient inbox messages improved mental task load and work exhaustion, reducing burnout scores for medical professionals. However, no improvements in overall response time were noted [42]. The study also showed that gastroenterology and hepatology nurses had higher draft utilization, a trend towards time saved, and positive net promoter scores, suggesting that future Gen AI tools may vary according to specific practice patterns and workflows [43]. These findings underscore the potential positive impact of Gen AI in healthcare settings to enhance efficiency and reduce burnout among physicians and other healthcare professionals.

Another study found that lower satisfaction and higher frustration with Electronic Health Records (EHRs) were significantly associated with physician burnout [44]. Gen AI has the potential to not only reduce the documentation burden but also remove the computer from the physician-patient interaction and bring the “care” back to healthcare [45]. This allows the physician to assess the patient’s situation quickly while the AI summarizes and surfaces the relevant data. Companies like Axtria are using Gen AI to improve patient and physician care and advance core information management capabilities such as master data and multi-modal data [46].

### Nursing

Gen AI demonstrates the potential to enhance patient care, optimize workflows, and advance nursing education and training. AI-enhanced simulations are increasingly integrated into nursing education, utilizing AI technologies to create realistic scenarios that replicate complex medical situations. This enables nursing students to acquire practical skills in a controlled environment, mitigating the risks associated with real-world scenarios. A recent review underscores the potential of AI in medical education, highlighting its ability to tailor learning experiences and refine clinical decision-making skills for healthcare professionals [47]. Other applications of AI systems include predicting patient fall risks, generating personalized treatment plans, and automating routine tasks such as medical documentation, allowing nurses more time for direct patient care [48].

AI-powered tools like voice-to-text transcription, automated charting, and intelligent data entry could reduce the time spent on documentation by 21–30%, saving nurses 95–134 h per year. Streamlining administrative processes like patient admissions, transfers, and discharges with AI could save nurses 37–46% of the time spent on these tasks, equating to 32–40 h per year [48]. This workflow optimization ensures efficient utilization of human resources, alleviating the burden on overburdened staff and potentially enhancing job satisfaction and retention [49].

Studies have revealed that AI chatbots can provide social support to patients, serving as a practical and accessible means of improving patient well-being, particularly in situations where traditional interventions are limited, such as during pandemics [50]. A recent study explores the transformative potential of Gen AI in nursing practices in Taiwan, focusing on the capabilities of the A + Nurse system, a digital assistant designed to automate routine nursing tasks, enhance communication within healthcare teams, and integrate current medical information into nursing workflows [51]. Prompt engineering is critical to effectively interact with AI, as precise prompts yield desired outputs [52]. Educators can leverage Gen AI-based models like ChatGPT to enrich teaching and learning experiences, including creating virtual simulation scenarios, developing lesson plans, and designing assessments [53].

### Synthetic Data Generation

Synthetic data, as defined by the Royal Society and The Alan Turing Institute, is data that has been generated using a purpose-built mathematical model or algorithm to address a data science task [54]. GANs and VAEs, among other technologies, can be used to generate synthetic data that safely replaces sensitive patient information while complying with strict privacy regulations such as GDPR and HIPAA [55–57]. Generated through generative models, synthetic data has numerous applications, including AI model training, validation, realistic simulations for medical training, and decision support systems, and enables researchers to conduct large-scale studies and analyses without real patient data [58]. Synthetic EHR and imaging data, developed using GANs, aid research and enhance diagnostic algorithms without compromising patient privacy [59].

A recent study describes how synthetic healthcare records can be generated using convolutional GANs and convolutional autoencoders (CorGAN). This architecture captures correlations between adjacent medical features and addresses the challenge of generating discrete data outputs [60]. Synthetic data, when produced using techniques like Gaussian Copulas (GC), Conditional Generative Adversarial Networks (CGAN), VAEs, and Copula-GAN, can supplement real datasets, enhancing the performance and robustness of non-invasive diabetes prediction models [61]. While synthetic data generation is useful in medical tasks, data is often scarce to train robust predictive models. For instance, a 2023 study demonstrated that augmenting real datasets with synthetic chest radiograph images generated by latent diffusion models can improve classification performance [62].

Additionally, another recent study showed that tailored prompts provided to ChatGPT can produce task-specific synthetic data, substantially improving performance in tasks such as biological named entity recognition and relation extraction [63]. Synthetic data is invaluable for training models and analysis and in scenarios where data is missing from healthcare databases, potentially skewing clinical decisions [64]. MDClone, a startup in this field, offers a platform that provides synthetic health data, eliminating the need for direct access to patient records while ensuring regulatory compliance [65].

### Medical Image Analysis

Medical image analysis can be defined as the process of examining medical images for diagnosis, improved treatment planning, and disease monitoring. This analysis plays a pivotal role in fields such as radiology, neurology, and oncology, where examining large volumes of images and data is crucial for extracting significant information [66]. Gen AI synthesizes, augments, and interprets heterogeneous complex images across various modalities, such as X-rays, MRIs, and CT scans. AI models such as Generative Adversarial Networks (GANs) and Variational Autoencoders (VAEs), among several others, facilitate the synthesis of anatomically precise images for training and education, addressing the shortage of certain types of images and the subtleties of ethical and privacy issues [67]. GANs can synthesize MRI brain images specifically to enhance the detection accuracy of neurological disorders using machine-learning models without violating patient privacy regulations [67].

Furthermore, studies have shown that GANs can be used for data augmentation in liver lesion detection, where synthetic images improve the diagnostic accuracy of the convolutional neural networks (CNNs) that utilize these images [68]. The CGAN model, enhanced with a 3D discriminator and adaptive identity loss, significantly improves synthetic MRI generation, especially in studying Alzheimer’s disease progression. This addresses the limitations of previous models that primarily operate in 2D and suffer from spatial artifacts [69]. Gen AI is also highly effective for anomaly detection and segmentation, which are crucial for early disease detection and intervention. For instance, developing the AnoGAN model for segmenting anomalies in retinal images aids in early diabetic retinopathy diagnosis [71].

A study investigating the use of GAN to segment MRI images of the brain showed that the GAN technique outperformed traditional methods, achieving an average Dice coefficient of 0.89 [70]. Another study utilizing GAN to generate synthetic images of the retina found that these images were as effective as real images in training deep-learning models for diabetic retinopathy detection [71]. GANs are also employed in image enhancement and reconstruction, such as enhancing low-dose CT images to promote safer imaging practices with reduced radiation exposure [72]. Gen AI has the potential to not only reduce healthcare disparities through improved diagnostic accuracy but also to usher in a new era of personalized medicine, where patient-specific models will aid in surgical planning and make treatments more individualized [73].

### Population Health Management

Gen AI can play a crucial role in predicting health trends, personalizing medicine, and optimizing healthcare services by analyzing vast datasets and tailoring health interventions to meet service demands. A promising application of deep learning is to interpret biological data and make predictions of disease within populations, such as using neural networks to analyze genetic information for early disease detection. AI considerably brings value-based care for individual patients into practice by personalizing treatment plans and improving patient outcomes, contributing to improved population health [74, 75].

By Integrating individual-level health data with socio-markers(quantifiable social conditions)— AI enhances disease surveillance and predicts health risks more accurately [76]. Using mobile technologies and the Internet of Things (IoT) allows for real-time health monitoring. It enhances the ability of health systems to deliver care remotely, which is crucial during events like the Ebola outbreak [77]. For example, AI algorithms can analyze individual health data alongside socioeconomic factors to predict which populations are mostly at risk for certain diseases, allowing for targeted interventions and resources. In addition, IoT devices like wearable fitness trackers can provide continuous health monitoring for individuals in remote areas, enabling healthcare providers to remotely monitor and treat patients during emergencies such as disease outbreaks [78].

Gen AI can streamline service delivery and operations for public health and community health workers by reducing administrative burdens, optimizing supply chain processes, and fostering tailored interactions with diverse communities [79]. AI models excel at analyzing extensive and varied datasets to forecast outcomes like hospital readmissions and recommend proactive measures for patient well-being [80]. Further, by categorizing populations based on risk factors, AI models enhance the effectiveness of interventions by targeting high-risk groups. In addition, AI can help predict the population’s health needs, optimizing resource allocation. Health Catalyst and ClosedLoop.ai also focus on using the potential of AI for predictive analytics and population health management, thereby personalizing individual care plans for members [81]. As Gen AI continues to evolve, it is poised to pioneer a new population health era by harnessing a wide range of data sources, including real-time health data from wearable technology, to deliver increasingly precise interventions and drive enhanced health outcomes for entire populations.

### Risk Prediction of Pandemic Preparedness

The outbreak of the COVID-19 pandemic, coupled with rapid advancements in AI and ML algorithms, has paved the way for significant investments and developments in AI and ML for pandemic management and surveillance [82, 83]. Examples of AI use during the pandemic include disease forecasting, enhancing outbreak detection, and monitoring disease progression [84]. Gen AI processes real-time data from diverse sources, including social media, health reports, and environmental data, to identify early signs of emerging infectious diseases. Detecting unusual patterns or clusters can contribute to early warning systems [85]. AI has also supported public health initiatives through public policy efforts [86].

Additionally, socioeconomic factors have been modeled to gain improved insights into pandemic management [87]. A recent application of AI during the COVID-19 pandemic, spanning from 2020 to as recent as 2023, involves NERVTAG, a UK Government Advisory Group. They developed the QCOVID3 risk prediction algorithm to anticipate potential hospitalizations and deaths among those infected with COVID-19. Since this algorithm was implemented in Wales, UK, and its results were based on that area’s demographics, it can only be considered successful within that region [88]. Similarly, the Australian government developed a COVID-19 Pandemic Vulnerability Index (CPVI) to assess vulnerability across different areas in Australia, thereby helping to delay the pandemic’s exponential growth and mitigate its impact on communities and the healthcare system. This widespread experiment covered 563 zones, each with an average population of 45,000, encompassing the entire country. Australia’s CPVI evaluates vulnerability across four subsections: socio-demographics, medical conditions, public works, and land use patterns. By analyzing these factors, the index provides a more robust understanding of areas at risk during a pandemic [89]. Both NERVTAG’s algorithm and Australia’s CPVI have been successful in their respective regions. However, both relied on ample data and specific factors differentiating them from other COVID-19 tools. Researchers have also explored whether solely comorbidity data, socioeconomic status, age and race could predict COVID-19 death rates.

To determine how these factors relate to hospitalization, ICU admission, ventilator use, and mortality, researchers analyzed a dataset of 1.4 million diagnosed COVID-19 cases [90]. During the QCOVID3 risk prediction experiments, scientists found that the predictions closely matched actual numbers for deaths and admissions up to the fifteenth predicted risk level, beyond which the predictions for deaths were higher and lower for admissions [88]. In the CPVI experiment, the index indicated that vulnerability to COVID-19 is highest in more populated areas, whereas it is much lower in rural areas [89]. However, graphs based solely on comorbidity proved insignificant, as the comorbidity-predicted death rates were far from actual outcomes in the experiment in the US and other selected countries.

## Non-Clinical Applications

### Medical Education and Training

Gen AI has the potential to enhance medical education by producing realistic medical images and simulating patient interactions. [91] It allows students to explore a variety of medical conditions, including rare diseases and complex clinical cases, through detailed images. Advanced frameworks such as Generative Adversarial Networks (GANs) and Variational Autoencoders (VAEs) facilitate synthesizing these educational tools, thereby expanding the range of learning materials available to students. GANs, for example, can generate synthetic medical images for educational purposes, enriching the pool of diverse and essential learning materials [92]. Another critical benefit of Gen AI in medical education is its potential to improve machine translation, fostering knowledge exchange and global collaboration. The advent of Gen AI technology has enhanced the accuracy and sophistication of machine translation. For instance, eBay’s Machine Translation service demonstrates a 7% increase in translation accuracy over its previous iterations, highlighting the potential of AI to overcome language barriers [93].

Additionally, Gen AI models have been utilized in medical education and surgical training to create dynamic, interactive models of patient anatomy, allowing students to gain theoretical and practical experience without relying on real patients [94]. Large Language Models (LLMs) are invaluable in medical research, literature review, and writing because they can quickly extract accurate data with appropriate usage of terminologies and language [95]. LLMs like GPT-4 can provide personalized learning experiences through adaptive educational content tailored to learners’ needs. The increase in dermatological datasets with synthetic images has improved the accuracy of AI model classifications of skin diseases, making them effective and valuable tools in medical education [96]. Gen AI has the potential to personalize education by adapting content according to students’ performance and preferences, showcasing AI’s ability to cater to diverse learning needs [97]. Western Michigan University has enhanced its medical curriculum with over 100 h of simulation training, providing realistic patient scenarios and feedback from professors to prepare students for real-world medical situations [23]. As Gen AI technology continues to evolve, its application in medical education is expected to facilitate more advanced simulations and personalized learning pathways, preparing professionals for the dynamically changing field of medicine.

### Revenue Cycle Management

Gen AI integrated with Health Revenue Cycle Management (RCM) automates and enhances various sectors in healthcare financial operations, including automated data entry, patient communication, documentation of lab procedures, compliance, procurement, contracting, and fraud detection [98]. Utilizing AI for patient scrutiny and treatment records facilitates the automatic generation of accurate billing codes with fewer errors, ensuring compliance with frequently changing regulations [99]. Moreover, Gen AI can predict personalized patient communication strategies, offer predictive analytics for financial planning, and provide insights into preventive measures to reduce claim denials. Automating administrative tasks like coding and billing significantly reduces the workload for healthcare staff, allowing them to focus more on patient care [100]. Leveraging AI-generated insights to identify and address the root causes of claim denials can lead to more efficient and accurate RCM processes [101].

According to a study by the Deloitte Center for Health Solutions, Gen AI can save between 41% and 50% of the time spent by revenue cycle professionals across all revenue cycle stages, including patient access, clinical revenue cycle, and patient financial services [48]. For example, Codametrix and Adonis.io exemplify the application of advanced technologies in healthcare RCM. Codametrix Inc. uses Natural Language Processing (NLP) to extract data from unstructured medical texts. It transforms it into structured data for medical language interpretation, thus facilitating analysis, billing, and population health management [102]. Adonis.io integrates revenue intelligence and automation throughout the process, enhancing efficiency and effectiveness in healthcare RCM [103].

### Healthcare Marketing and Public Relations

Gen AI can assist healthcare organizations with marketing and public relations (PR) by personalizing content, automating customer support, and identifying areas for optimization in marketing campaigns [104]. By analyzing large datasets, Gen AI can create customized content, such as health tips and articles, that promote engagement between patients and healthcare providers [105]. It enhances personalized patient service by fine-tuning responses based on individual needs, supporting live agents, and providing real-time feedback, leading to higher customer satisfaction, faster issue resolution, and lower costs. Additionally, Gen AI can optimize supply chains by predicting disruptions, enabling scenario analysis, and assisting in supplier evaluations, allowing quicker adaptation to market changes and improved operational efficiency [13]. Evidence indicates that one-third of individuals in the United States first search online for health information before seeking professional medical advice [106].

Research shows a rising trend in individuals investigating mental health symptoms online, with mental health queries increasing significantly [107]. The development of digital mental health tools, such as AI-driven chatbots,  has escalated to meet this growing demand. This highlights the critical importance of enhanced emotional intelligence in general AI systems, especially in providing empathetic and personalized user interactions. Generative AI revolutionizes strategic content analysis in marketing by identifying high-performing content types and messaging strategies [108]. The integrative Gen AI-equipped tools add competitive advantages to health organizations in patient engagements and public interactions, underlining its transformational potential for health marketing and PR [109].

## Conclusion

This paper has explored the diverse impacts of Gen AI in both clinical and non-clinical healthcare domains. Applications such as personalized treatment plans, medical image analysis, and synthetic data generation have demonstrated the transformative capabilities of Gen AI in enhancing diagnostic accuracy, streamlining operations, and facilitating personalized medicine. Furthermore, integrating Gen AI in medical education, healthcare marketing, and revenue cycle management illustrates its potential to revolutionize learning experiences, optimize financial operations, and enhance patient engagement strategies. It is also critical to recognize the potential to augment and improve the performance of human experts in healthcare using personal adaptivity with generative AI. To ensure the continued advancement of Gen AI in healthcare, it is imperative to establish robust ethical guidelines and governance frameworks that address data privacy, algorithmic bias, transparency, and accountability. By promoting and strengthening a collaborative environment among researchers, clinicians, policymakers and prioritizing ethical considerations, Gen AI can continue to be a significant force for innovation and improvement in healthcare services worldwide.

## Data Availability

No datasets were generated or analysed during the current study.
